# Two new species of *Paraboea* (Gesneriaceae) in *Caryota obtusa* forests in Southwest China, with compound and simple dichasia, respectively

**DOI:** 10.3897/phytokeys.157.32534

**Published:** 2020-08-26

**Authors:** Yu-Min Shui, Shi-Wei Guo, Li Chen, Wen-Hong Chen

**Affiliations:** 1 CAS Key Laboratory for Plant Diversity and Biogeography of East Asia, Kunming Institute of Botany, Chinese Academy of Sciences, 132 Lanhei Road, Kunming 650201, Yunnan, China Kunming Institute of Botany, Chinese Academy of Sciences Kunming China; 2 Karst Conservation Initiative of Yunnan, Kunming 650201, Yunnan, China Karst Conservation Initiative of Yunnan Kunming China; 3 School of Life Sciences, Yunnan University, Kunming 650091, Yunnan, China University of Chinese Academy of Sciences Beijing China; 4 University of Chinese Academy of Sciences, Beijing 100049, China Yunnan University Kunming China

**Keywords:** Dichasia, Karst regions, new species, *Paraboea brevipedunculata*, *Paraboea myriantha*

## Abstract

Two new species of Gesneriaceae, *Paraboea myriantha***sp. nov.** and *P. brevipedunculata***sp. nov.** are described and illustrated with photos. They grow in the *Caryota obtusa* forests from Yunnan Province of China. *P. myriantha* is closely related to *P. glutinosa* (Hand.-Mazz.) K.Y.Pan, but differs mainly in corolla outside glandular-puberulent, adaxial corolla lobes semicordate, corolla tube obliquely campanulate, and filaments glandular-puberulent. *P. brevipedunculata* is closely related to *P. crassifolia* (Hemsley) B. L. Burtt, but different mainly in simple dichasia with 1 and 2 flowers, peduncles 0.5–2 cm long and capsules slightly twisted. The geographical relationship between the two new species and their similar species has been discussed.

## Introduction

The genus *Paraboea* (Clarke) Ridl. (Gesneriaceae), including about 90 species, mainly occurs in Bhutan, China, Indonesia, Malaysia, Myanmar, Philippines, Thailand and Vietnam (Xu et al. 2008). Recently, several new species have been published (Chen et al. 2008, 2012; Kiew 2010; Xu et al. 2012; Wen et al. 2013; He et al. 2018). Most of them are distributed in the karst regions in South China and Indo-China (Li 1987; Wang 1990; Xu 1993; Fang et al. 1995; Zhu et al. 2003; Li and Wang 2004; Shui and Chen 2006; Zhu 2007; Shui et al. 2017). The genus is easily recognised by the thick hairs on the adaxial surface and lax hairs on the abaxial surface of the leaves in the karst regions, especially in *Caryota obtusa* forest in southwest China (Chen et al. 2017). The forest is a special vegetation subtype in the karst regions and harboured numerous endemic species, such as *Paraboea hekouensis* Y.M. Shui & W.H. Chen and *P. manhaoensis* Y.M. Shui & W.H. Chen in Gesneriaceae (Chen et al. 2012; Chen et al. 2019).

**Figure 1.**
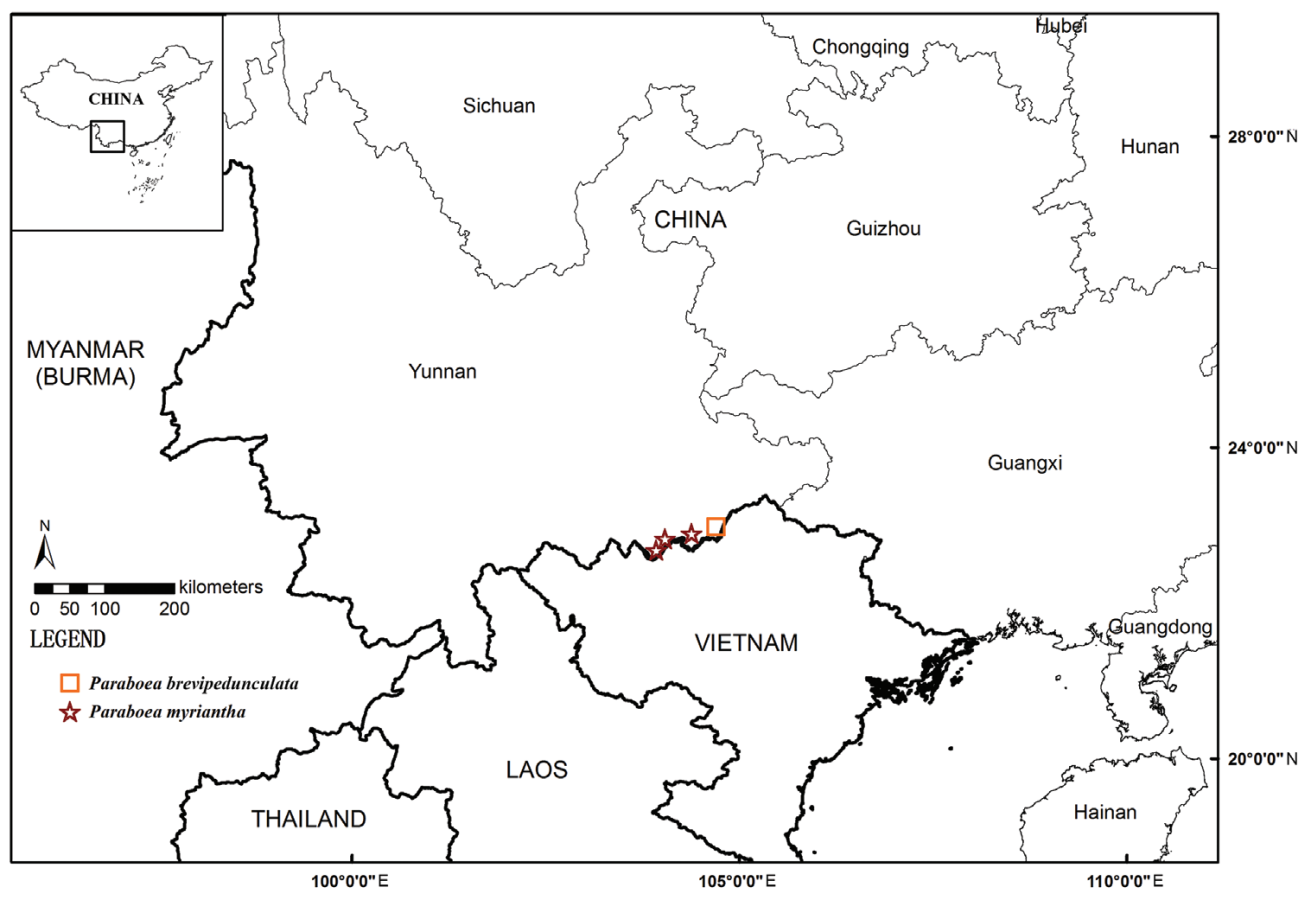
The geographical distribution of *Paraboea brevipedunculata* W.H. Chen & Y.M. Shui, sp. nov. (square) and *P. myriantha* Y.M. Shui & W.H. Chen, sp. nov. (star).

Long-term surveys of *Caryota* forests revealed some new findings in the karst regions in Southwest China. From 2001 to 2005, during our botanical exploration to *Caryota* forests in karst areas in the southeast of Yunnan Province, China, we collected some species of the genus *Paraboea* in Gesneriaceae in Hekou County of SE Yunnan, China (Figure 1). Amongst them, one species with up to 0.9 m tall habit, produces a compound dichasium with hundreds of flowers (Shui and Chen 2006; Chen et al. 2008). With further observation, it is similar to *P. glanduliflora* Barnett in glandular hairs outside the corollas and different in the basal leaves (Wang et al. 2012). After careful comparison with the other species of *Paraboea* in China (Li 1987; Wang 1990; Fang et al. 1995; Li and Wang 2004; Chen et al. 2008, 2012, 2017; He et al. 2018) and bordering countries (Thúợngtiền 2000; Xu et al. 2008), we confirmed that the species represents an undescribed species of *Paraboea* in Gesneriaceae. After a complete examination to the main worldwide herbaria, we confirmed several additional specimens collected in the adjacent karst regions in China during the 2001–2018 period.

In June 2013, on the other hand, we collected one small doubtful species of *Paraboea* with fruits in Malipo county in the southeast of Yunnan Province (Figure 1). In the field, it grows on cliffs, as well as in the *Caryota* forest at the border with Vietnam in Malipo county, Yunnan, China. However, we missed the flowering period in 2014 and 2015. In May 2016, we collected the plants with flowers and confirmed that it belonged to the genus *Paraboea*. After an examination of literatures and related specimens, we determined that it is unique in ca. 5 cm high habit and simple dichasia and should be an undescribed species in the genus. It is possible that it may be collected in Vietnam in the future (Figure 1).

## Materials and methods

We confirmed two new species after examination of the specimens preserved in worldwide herbaria (E, IBSC, K, KUN, P, PE). We took photographs of the habit and macro-morphological characters in the field. Subsequently, we carried out morphological observations and measurements of the two new species, based on living plants in the field and Kunming Botanical Garden, together with additional specimens in KUN. All micro-morphological characters were observed and photographed with a Leica S8 APO stereomicroscope and a Nikon D700 microscope camera.

## Taxonomy

### Paraboea myriantha

Taxon classificationPlantaeLamialesGesneriaceae

Y.M. Shui & W.H. Chen sp. nov.

1D235CCE-C6BC-575B-9E66-7B22C2259765

urn:lsid:ipni.org:names:77211197-1

Figure 2

#### Type.

China. Yunnan Province: Hekou County, Nanxi Community, 22°38'18.44"N, 104°00'28.93"E, in the limestone forests, alt. 900 m, 26 August 2005, in flowers, *Y.M. Shui et al. 44536* (holotype KUN).

#### Diagnosis.

The new species is similar to *P. glutinosa* (Hand.-Mazz.) K.Y.Pan in winged petioles, leaf-like bracts and compound dichasia, but distinguished by adaxial corolla lobes semicordate (vs. nearly rounded), tube obliquely campanulate (vs. urceolate) outside glandular-puberulent (vs. glabrous or rarely pubescent) and laterally uneven (vs. even), and glandular-puberulent filament (vs. covered by a beard of multicellular hairs); and similar to *P. thorelii* (Pellegr.) B.L.Burtt in winged petioles and compound dichasia, but distinguished by corolla tubes 9–10 mm long (vs. 3–4 mm long) outside glandular-puberulent (vs. glabrous) and staminodes 2 (vs. inconspicuous).

#### Description.

Herbs, up to 90 cm tall, stems 10–20 cm. Leaves opposite, clustered at stem apex; petiole 5–8 (–15) cm long and winged, wings (1–) 5–20 mm wide each side; leaf blade obovate, 12–30 (–48) × 5–13 (–16) cm, chartaceous, adaxially sparsely pubescent to glabrous, abaxially densely matted arachnoid, base cuneate to wing-like on petiole, margin repand-crenate and involute, apex mucronate to rounded; midrib depressed adaxially, protuberant abaxially; lateral veins 8–13 (–16) pairs, obscure adaxially and distinct abaxially. Dichasium terminal paniculate, with hundreds of flowers; peduncles up to 70 cm, densely arachnoid when young, sparsely puberulent to glabrous when mature; bracts (the lowermost fertile leaves) 2, leaf-like, ca. 7 × 3 cm, densely arachnoid abaxially; bracteoles 2, linear, ca. 2 × 0.5 mm; pedicels 5–8 mm long. Calyx 5-sect from base; segments linear, ca. 1 mm long, glabrous. Corolla purple adaxially and white abaxially (corollas purple when young), outside glandular-puberulent; tube obliquely campanulate, ca. 10 mm long, outside glandular-puberulent; adaxial lip 2-lobed, lobes semicordate ca. 3 × 6 mm; abaxial lip 3-lobed, lobes ca. 5× 6 mm. Stamens 2, included; filaments lateral-fixed, ca. 8 mm long, glandular-puberulent from middle to top; anthers ca. 5 mm long; staminodes 2, 3–5 mm long. Pistil glabrous; ovary oblong, ca. 6 mm long; style linear, 4–5 mm long; stigma capitate. Capsule obviously twisted, 4.5–6.7 cm long, glabrous, with persistent calyx. Seeds ellipsoid, 0.6–0.7 × 0.15–0.2 mm.

#### Etymology.

The new species is named after its numerous flowers per an individual.

#### Distribution and habitat.

The new species only grows in *Caryota obtusa* forests of limestone areas in Hekou and Maguan counties of Yunnan, China (Figure 1).

#### Phenology.

Flowering from June to August; fruiting from July to November.

#### Additional specimens examined (paratype).

China. Yunnan Province: Hekou County, in dense forests on the limestone hillsides, alt. 700–950 m, 21 October 2001, *Y.M. Shui et al. 15105* (KUN); at the same county, in the limestone seasonal forests, alt. 1000–1200 m, with fruits of last year, 28 March 2002, *Y.M. Shui et al. 20595* (KUN); at the same county, in dense forests on limestone hillsides, alt. 1000 m, with young dichasia, 28 March 2002, *Y.M. Shui et al. 20946* (KUN); at the same county, Nanxi Zhen, 22°40'8"N, 104°01'16"E, in forests, alt. 900 m. 6 September 2013, *Y.M. Shui, B. Xiao, J. Wang et al. B2013-528* (KUN). Maguan County, Gulinqing Community, 22°43'N, 103°59'E, in the evergreen broad-leaved forests on limestone hillsides, alt. 1000 m altitude, 3 October 2002, *Y.M. Shui et al. 30261* (KUN); at the same county, 3 October 2002, *Y.M. Shui et al. s.n.* (KUN); at the same county, in the limestone evergreen broad-leaved forests, alt. 794 m, 11 November 2006, *Y.M. Shui et al. 16118* (KUN); at the same county, Jiahanqing Community, Moshizhai Village, 9 August 2018, *Y.M. Shui et al. B2018-183* (KUN).

#### Note.

**Figure 2.**
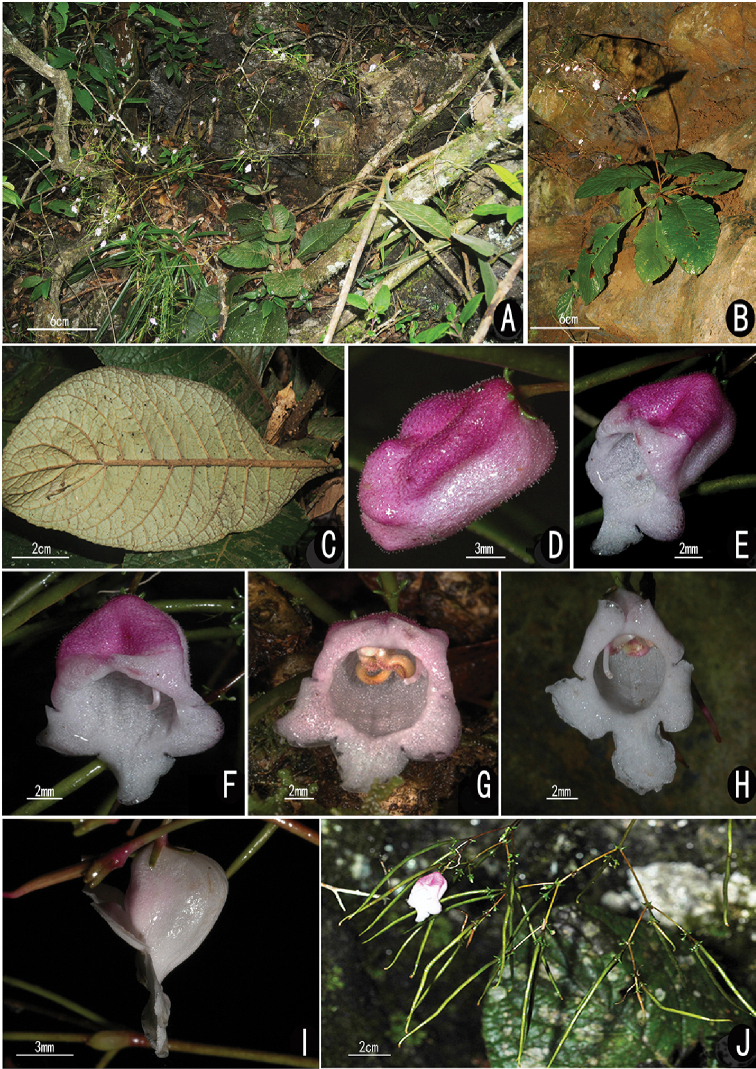
*Paraboea myriantha* Y.M. Shui & W.H. Chen, sp. nov. (**A, C–G, J**) and its similar species *P. glutinosa* (**B, H, I**) **A, B** habitat **C** abaxial surface of leaf **D** flower bud **E** lateral view of flower **F** bird view of flower **G, H** front view of flower **I** lateral view of flower **J** flower and fruits (**A** and **C** photographed by Gui-Liang Zhang and all the others by Yu-Min Shui).

The new species appeared as *Paraboea auriculata* Y.M. Shui & W.H. Chen (nom. nud.) because of its winged petioles in Shui and Chen (2006). However, we decided to name it as *Paraboea myriantha*, after careful comparison of references and type specimens with similar species, *P. glutinosa* (Hand.-Mazz.) K.Y.Pan and *P. thorelii* (Pellegr.) B.L.Burtt. The new species is similar to the above two species on winged leaves and compound dichasia, but distinguished by corolla lobes (shape) and tubes (shape and indumenti) and glandular filament, which are described in diagnostics (Figure 2; Xu et al. 2008; Vu et al. 2011). Furthermore, *P. glutinosa* is distributed in South China, *P. thorelii* in South Laos (type locality) and North Vietnam, but the new species we proposed seems geographically distributed between the above two similar species. So, the future molecular work in the context of the whole genus may reveal if the above three species become a species complex with obvious geographical replacement.

### Paraboea brevipedunculata

Taxon classificationPlantaeLamialesGesneriaceae

W.H. Chen & Y.M. Shui sp. nov.

32014FB2-62CE-5BB2-82F9-29CEEDE9347F

urn:lsid:ipni.org:names:77211198-1

Figure 3

#### Type.

China. Yunnan province: Malipo County, Tianbao community, 22°58'33.31"N, 104°50'32.92"E, in limestone forests, alt. 900 m, 30 April 2017, *Y.M. Shui & W.H. Chen B2017-1342* (holotype, KUN).

**Figure 3.**
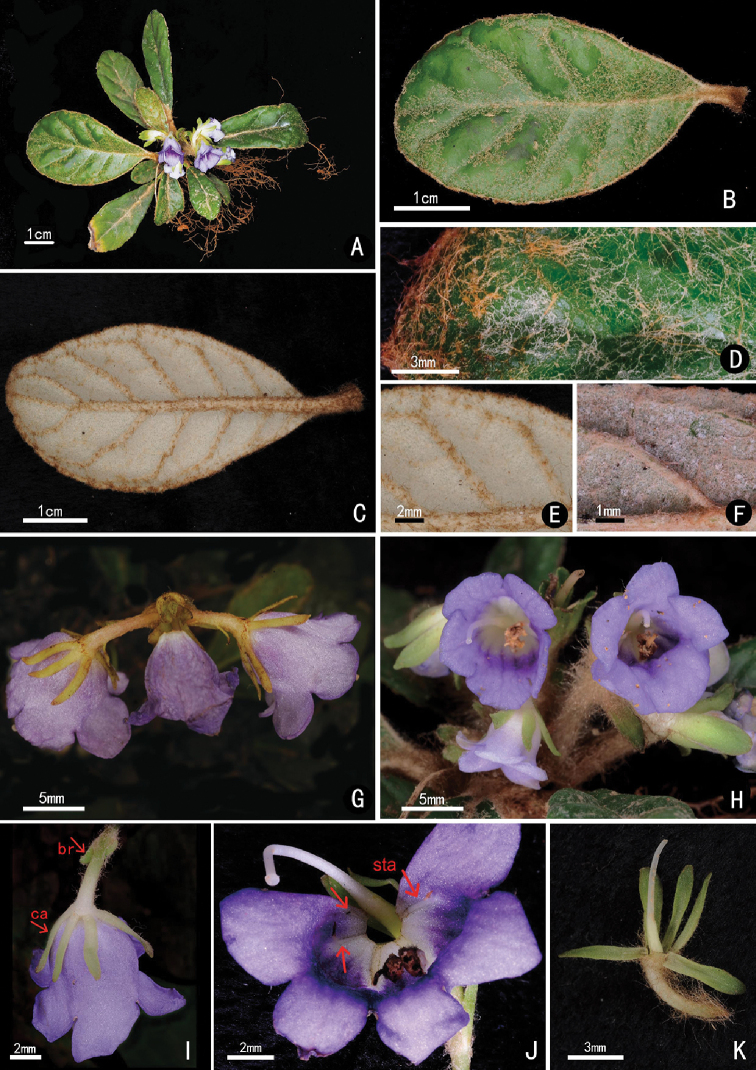
*Paraboea brevipedunculata* W.H. Chen & Y.M. Shui, sp. nov. **A** habit **B** adaxial surface of leaf **C** abaxial side of leaf **D** adaxial surface of leaf, showing indumentum **E, F** abaxial surface of leaves, showing indumenti **G** dichasia **H** front view of flower **I** vertical view of flower, showing bracteoles (br) and calyx lobe (ca) **J** opened corolla, showing staminodes (sta) and pistil **K** pistil with calyx lobes (All photographed by Yu-Min Shui).

#### Diagnosis.

*Paraboea brevipedunculata* is similar to *P. crassifolia* (Hemsley) B. L. Burtt in morphology and indumenti of the leaves, but different in simple dichasia with 1–2 flowers (vs. compound dichasia with many flowers), peduncle 0.5–2 cm (vs. 8–12 cm), 4–5 mm calyx segments (vs. 2–3 mm), capsules slightly twisted (vs. multi-twisted) and 0.6–0.7 mm long when mature (2–2.5 cm long). The new species is also similar to *P. velutina* (W.T.Wang & C.Z.Gao) B.L.Burtt. in the small plant, short peduncle and simple dichasia, but distinguished by purple corolla (vs. white), calyx 4–5 mm long (vs. ca. 1 mm), lobes of adaxial lip ca. 3 × 5 mm (vs. ca. 1.5 × 1 mm), lobes of abaxial lip ca. 5 × 7 mm (vs. ca. 1.5 × 2.3 mm) and capsule slightly twisted (vs. not twisted).

#### Description.

Herbs 4–5 cm high and without stems. Leaves clustered; petiole very short, 0.2–1 cm, densely arachnoid; leaf blade obovate, 2.6–6 × 1–3 cm, thick papery, adaxially pubescent when young and subglabrous when mature, abaxially densely matted arachnoid, base cuneate, margin subentire to shallowly repand-crenate, apex rounded; midrib depressed adaxially, protuberant abaxially; lateral veins 4–7 pairs, obscure adaxially and distinct abaxially. Dichasium terminal axillary, with 1–4 flowers; peduncle 0.5–2 cm, densely arachnoid; bracts 2, 0.5–0.6 × ca. 0.1 cm, sparsely pubescent abaxially; bracteoles 2 (sometimes absent), linear, ca. 2 × 0.5 mm; pedicel 0.3–1.2 cm long, densely arachnoid. Calyx 5-sect from base; segments linear, 4–5 × 1–2 mm, glabrous. Corolla purple, glabrous; tube 5–7 mm; adaxial lip 2-lobed, lobes ca. 3 × 5 mm; abaxial lip 3-lobed, lobes ca. 5× 7 mm. Stamens 2, included; filaments curved, ca. 4 mm long, glabrous; anthers ca. 2 mm long; staminodes 3, the lateral two ca. 1 mm long and the middle one ca. 0.5 mm long. Pistil glabrous; ovary oblong, ca. 2 mm long; style linear, 6–7 mm long; stigma capitate. Capsule 0.6–0.7 mm long when mature, slightly twisted, glabrous, with persistent calyx. Seeds ellipsoid, 5–7 × 0.2–0.3 mm.

#### Etymology.

The new species is named after its short peduncle per dichasium.

#### Distribution and habitat.

The new species only grows in *Caryota obtusa* forests of limestone areas in Malipo County of Yunnan, China (Figure 1).

#### Phenology.

Flowering from April to May; fruiting from June to July.

#### Additional specimens examined (paratype).

China. Yunnan Province: Malipo County, 22°58'33.31"N, 104°50'32.92"E, in limestone forests, alt. 900 m, 24 June 2013, *Y.M. Shui & W.H. Chen B2013-094* (KUN); Malipo County, Tianbao, 22°58'33.31"N, 104°50'32.92"E, in limestone forests, alt. 900 m, 30 April 2017, *Y.M. Shui & W.H. Chen B2017-1342* (KUN); the same place, 14 September 2018, in fruits, *Y.M. Shui & W.H. Chen B2018-021* (KUN).

#### Note.

The new species is more similar to *P. crassifolia* than *P. neurophylla* (Collett & Hems1.) B.L. Burtt in its linear bracts (Wang et al. 1998; Li and Wang 2004). *P. crassifolia* is distributed in W Huibei, SE Chongqing, Guizhou, Guangxi and SE Yunnan in China, while *P. neurophylla* is distributed in China (Central and West Yunnan) and Myanmar. The new species is distributed in SE Yunnan and shares the similar distribution with *P. crassifolia*, which is considered as the similar species to the new species. Besides, as to the small habit and fruits, it is somewhat similar to *P. velutina* in West Guangxi, which is next to SE Yunnan, but distinguished by corolla colour, size of calyx and corolla lobes, and twisted capsules (see the above diagnosis).

Vu et al. (2011) reported *Paraboea neurophylla* as a new record in Vietnam. The voucher specimens are collected from Ba Be National Park, Bac Kan province, Vietnam. However, the figure (based on *HLF 608* in HN) reveals that it seems to be conspecific with the new species we proposed here. Additionally, the description and geographical distribution of the new record in Vietnam roughly match that of the new species (Vu et al. 2011). Although we are still waiting for further confirmation from the detailed surveys, it is possible that the new species will also occur in North Vietnam. In fact, *P. neurophylla* grows at ca. 2000 m elevation in China (Yunnan, e.g. *B.Y. Qiu 55121* in PE, *P. I Mao 1322* in PE, *S. E. Liu 831, 13970, 14087, 19713 and 20886* in PE, *K.M. Feng 10115* in PE, *K.Y. Pan 1* in PE, *J. Wu WJ2015010* in PE, *Z.J. Qiu QZJ-0936* in PE, *C.J. Chen 38* in PE, *J.S. Xin 51404* in IBSC) and Myanmar (Shan hills, *Collett 804*, holotype K and isotype in E), but its habitat is very different from the habitat of the new species at less than 1000 m elevation (Wang 1990; Wang et al. 1998; Xu et al. 2008).

## Supplementary Material

XML Treatment for Paraboea myriantha

XML Treatment for Paraboea brevipedunculata
